# Supplementary material for: Insights from DNA Barcodes-Based Phylogenetic Analysis of Medicinal Plants and Estimation of Their Conservation Status: A Case Study in the Tianshan Wild Forest, China
Source: Plants (Basel). 2025 Jan 1;14(1):99. doi: 10.3390/plants14010099 (PMC11723300; doi:10.3390/plants14010099)
Supplement: Supplementary file 1 [file plants-14-00099-s001.zip › plants-3334060-supplementary.pdf]

Table S1 Species Voucher Collection Information

No.	Name	Collection Date	Specimen No.	Habitat
1	*Aconitum leucostomum*	2023/7/6	XY2023070602	Spruce undergrowth
2	*Aconitum monticola*	2023/7/1	QTPTS20200505	Subalpine grassland
3	*Amaranthus retroflexus*	2022/6/25	ZS202210145	Stony hillside gullies and riverbanks
4	*Androsace septentrionalis*	2023/7/13	DK2023071306	Coniferous meadows
5	*Aquilegia atrovinosa*	2023/7/6	XY2023070601	Subalpine grassland
6	*Arctium tomentosum*	2023/7/8	GL2023070813	Broadleaved forests
8	*Arnebia euchroma*	2020/7/5	DK202005310	Hillside scrub meadow
9	*Artemisia dracunculus*	2020/7/5	CX2022071963	Roadside gravel
10	*Artemisia tanacetifolia*	2023/7/13	DK2023071320	Subalpine grassland
11	*Aster alpinus*	2020/7/5	XY2023071210	Alpine rhyolite beach
12	*Avena fatua*	2022/6/25	ZS202210138	Riparian slopes
13	*Berberis iliensis*	2022/6/26	HC202210185	Coniferous Scrub Meadow
14	*Betula tianschanica*	2023/7/7	XY2023070720	River meadow
15	*Bistorta vivipara*	2023/7/12	XY2023071246	Subalpine grassland
16	*Bupleurum thianschanicum*	2023/7/7	XY2023070718	Gravelly slopes
17	*Bupleurum triradiatum*	2023/7/12	XY2023071224	Spruce understorey
18	*Campanula glomerata*	2020/7/1	QTPTS20200090	Subalpine meadow
19	*Campanula glomerata*	2020/6/21	QTPTS20200030	Subalpine meadow
20	*Campanula glomerata* subsp.*speciosa*	2023/7/8	GL2023070807	Subalpine meadow
21	*Cannabis sativa*	2020/7/4	qtpts1176	Subalpine meadow
22	*Caragana jubata*	2023/7/13	DK2023071313	Subalpine meadow
23	*Carpesium triste*	2023/7/8	GL2023070801	Subalpine Meadow
24	*Carum carvi*	2023/7/12	XY2023071211	Spruce Forest
29	*Clematis glauca*	2023/7/7	XY2023070706	Subalpine grassland
28	*Clematis tangutica*	2020/7/5	DK202005312	Riverbanks
30	*Codonopsis clematidea*	2023/7/7	XY2023070725	Coniferous meadows
31	*Conium maculatum*	2023/7/8	GL2023070803	Subalpine grassland
33	*Crataegus songarica*	2022/6/26	HC202210195	Coniferous Scrub Meadow
34	*Cynoglossum divaricatum*	2020/7/3	QTPTS20200578	Coniferous Forest Meadow
35	*Cynoglossum officinale*	2023/7/7	XY2023070710	Subalpine meadow
36	*Dasiphora fruticosa*	2023/7/12	XY2023071216	Subalpine Meadow
37	*Daucus carota*	2023/7/10	GL2023071020	Conifer Scrub Meadow
26	*Dichodon cerastoides*	2022/6/22	CX2022071921	Stony slopes
27	*Dichodon cerastoides*	2020/7/5	qtpts1234	Stony slopes
38	*Dictamnus dasycarpus*	2023/7/6	XY2023070603	Mixed coniferous and broadleaved forest downslope
39	*Doronicum altaicum*	2023/7/12	XY2023071201	Subalpine grassland
40	*Dracocephalum grandiflorum*	2023/7/13	DK2023071308	Subalpine grassland
41	*Dracocephalum nutans*	2022/6/26	HC202210183	Broadleaved coniferous forest scrub meadow
42	*Dracocephalum ruyschiana*	2023/7/7	XY2023070716	Subalpine grassland
43	*Elsholtzia densa*	2023/7/10	GL2023071010	Subalpine grassland
44	*Epilobium palustre*	2023/7/9	GL2023070918	River sand
45	*Epipactis helleborine*	2023/7/8	GL2023070806	Subalpine grassland
32	*Erigeron canadensis*	2022/6/24	XY202210120	Subalpine grassland
46	*Erysimum diffusum*	2022/6/26	HC202210200	Riverine alluvial beach
47	*Erysimum diffusum*	2020/7/4	qtpts1218	Riparian alluvial beach
48	*Erysimum flavum* subsp.*altaicum*	2023/7/13	DK2023071303	Riparian alluvial flats
49	*Gagea serotina*	2023/7/13	DK2023071330	Gravelly hillside meadows

50	*Glycyrrhiza uralensis*	2020/7/4	XY202210131	Subalpine grassland
51	*Glycyrrhiza uralensis*	2023/7/6	XY2023070609	Subalpine grassland
52	*Heracleum dissectum*	2023/7/9	GL2023070915	Forest clearings
53	*Humulus lupulus*	2022/6/26	HC202210193	Coniferous Scrub Meadow
54	*Hyoscyamus niger*	2023/7/9	GL2023070911	Subalpine grassland
55	*Inula britannica*	2023/7/10	GL2023071013	Subalpine grassland
56	*Inula racemosa*	2023/7/8	GL2023070824	Subalpine grassland
57	*Inula rhizocephala*	2023/7/8	GL2023070821	Subalpine grassland
95	*Juniperus sabina*	2022/6/22	DK202210005	Stony slopes
58	*Lamium album*	2023/7/12	XY2023071208	Spruce understorey
59	*Lathyrus pratensis*	2023/7/8	GL2023070814	Hillside woodland
60	*Lathyrus tuberosus*	2023/7/10	GL2023071009	Subalpine meadow
61	*Leontopodium leontopodioides*	2022/7/19	CX2022071906	Subalpine meadow
62	*Leontopodium leontopodioides*	2020/6/21	QTPTS20200079	Subalpine meadow
63	*Leonurus turkestanicus*	2023/7/7	XY2023070721	Subalpine grassland
64	*Ligularia alpigena*	2022/6/25	ZS202210166	Broadleaved understorey
65	*Ligularia heterophylla*	2020/6/25	XY2023071241	Subalpine grassland
66	*Lonicera hispida*	2023/7/7	XY2023070709	Subalpine grassland
67	*Malva pusilla*	2022/6/23	GL2023070921	Subalpine meadow
68	*Matricaria matricarioides*	2023/7/12	XY2023071232	Spruce understorey
69	*Medicago lupulina*	2022/6/24	CX2022071943	Coniferous meadows
70	*Myricaria bracteata*	2023/7/9	GL2023070905	Gravelly slopes and gullies
71	*Oreomecon nudicaulis*	2023/7/12	XY2023071240	Subalpine meadow
72	*Origanum vulgare*	2023/7/7	XY2023070715	Coniferous Scrub Meadow
73	*Orthilia secunda*	2023/7/12	XY2023071204	Spruce forest
74	*Oxyria digyna*	2023/7/13	DK2023071326	Stony slopes
75	*Oxytropis ochroleuca*	2023/7/12	XY2023071206	Subalpine meadow
77	*Parnassia palustris*	2023/7/12	XY2023071220	Subalpine meadow
78	*Phleum pratense*	2023/7/12	XY2023071228	Subalpine meadow
79	*Plantago media*	2022/6/25	ZS202210151	Broadleaf understorey
80	*Poa pratensis*	2020/7/6	qtpts1315	Hillside woodland
81	*Polemonium caeruleum*	2023/7/12	XY2023071213	Subalpine Meadow
82	*Potentilla chinensis*	2022/6/26	HC202210201	Coniferous Meadow
83	*Primula algida*	2022/6/22	DK2023071314	Stony slopes
84	*Primula algida*	2019/7/26	20190107	Stony mountain slopes
7	*Prunus armeniaca*	2022/6/26	HC202210194	Broadleaved understorey
76	*Prunus padus*	2020/7/1	ZS202210166	Coniferous Meadow
85	*Pseudolysimachion alatavicum*	2023/7/13	DK2023071304	Subalpine meadow
86	*Pyrola rotundifolia*	2023/7/12	XY2023071242	Subalpine Meadow
25	*Rhaponticoides ruthenica*	2020/7/1	QTPTS20200518	Subalpine grassland
87	*Rheum wittrockii*	2023/7/12	XY2023071229	Hillside Scrub Meadow
88	*Rhodiola kirilowii*	2023/7/13	DK2023071331	Subalpine grassland
89	*Rhodiola quadrifida*	2023/7/13	DK2023071327	Stony slopes
90	*Roemeria refracta*	2022/6/24	XY202210131	Arid Hillside Grassland
91	*Roemeria refracta*	2023/7/10	GL2023071003	Arid hillside meadow
92	*Rosa laxa*	2022/6/25	ZS202210157	Coniferous Forest Meadow
93	*Rubus idaeus*	2023/7/7	XY2023070708	Coniferous Scrub Meadow
94	*Rubus sachalinensis*	2020/6/29	202005158	Subalpine grassland
96	*Salvia deserta*	2022/6/24	CX2022071960	Broadleaved forest understory

97	*Salvia japonica*	2023/7/9	GL2023070908	Subalpine grassland
98	*Saxifraga cernua*	2023/7/13	DK2023071321	Alpine driftstone beach
99	*Sophora alopecuroides*	2020/7/3	QTPTS20200596	Stony hillside gullies
100	*Sorbus tianschanica*	2023/7/7	XY2023070719	Coniferous meadows
101	*Sorbus tianschanica*	2023/7/7	XY2023070719	Coniferous meadows
102	*Sparganium stoloniferum*	2023/7/9	GL2023070903	Forest puddle
103	*Thalictrum simplex*	2023/7/12	XY2023071215	Spruce forest
104	*Thymus marschallianus*	2023/7/9	GL2023070916	Subalpine meadow
105	*Thymus proximus*	2023/7/12	XY2023071221	Subalpine Meadow
106	*Triglochin palustris*	2023/7/9	GL2023070909	Forest puddle
107	*Valeriana officinalis*	2023/7/12	XY2023071234	Subalpine Meadow
108	*Verbascum thapsus*	2023/7/7	XY2023070714	Subalpine Meadow
109	*Vicia cracca*	2023/7/8	GL2023070805	Subalpine Meadow

Table S2 The sequences downloaded from NCBI for genetic distance analysis

No.	ITS	*matK*	*rbcL*	*ITS+matK*	*matK+rbcL*	*ITS+rbcL*	*ITS+matK+rbcL*
1	*Aconitum leucostomum* KY417299	*Aconitum leucostomum* MH195426	*Aconitum pseudolaeve* MN648400	*Aconitum loczyanum* LC456243	*Aconitum barbatum* MK253470	*Aconitum monticola* JF975813	*Aconitum loczyanum* LC456243
2	*Aconitum nemorum* MT924040	*Aconitum orientale* MZ044918	*Amaranthus retroflexus* MW646089	*Aconitum orientale* MZ044918	*Aconitum orientale* MZ044918	*Aconitum sinomontanum* JF975823	*Aconitum orientale* MZ044918
3	*Amaranthus retroflexus* KX079528	*Amaranthus retroflexus* MG685166	*Androsace septentrionalis* NC080503	*Amaranthus retroflexus* MG685166	*Amaranthus retroflexus* MG685166	*Amaranthus retroflexus* KX079528	*Amaranthus retroflexus* MG685166
4	*Androsace septentrionalis* MT924187	*Androsace septentrionalis* NC080503	*Aquilegia kansuensis* MT875877	*Androsace septentrionalis* NC080503	*Androsace septentrionalis* NC080503	*Androsace septentrionalis* NC080503	*Androsace septentrionalis* NC080503
5	*Aquilegia yabeana* MH808952	*Aquilegia pubescens* MF963615	*Arctium lappa* NC042724	*Aquilegia longissima* OL537805	*Aquilegia pubescens* MF963615	*Aquilegia ecalcarata* MH711080	*Aquilegia canadensis* HQ593174
6	*Arctium tomentosum* MT922632	*Arctium tomentosum* MG224843	*Arnebia euchroma* ON529958	*Arctium tomentosum* MG224843	*Arctium tomentosum* MG224843	*Arctium tomentosum* MT922632	*Arctium tomentosum* MG224843
7	*Arnebia euchroma* EF199848	*Arnebia euchroma* ON529957	*Artemisia rubripes* MG951496	*Arnebia euchroma* ON529957	*Arnebia euchroma* ON529957	*Arnebia euchroma* ON529958	*Arnebia euchroma* ON529957
8	*Artemisia chamaemelifolia* MH100697	*Artemisia dracunculus* ON641377	*Avena fatua* NC044170	*Artemisia dracunculus* var.*glauca* OR668915	*Artemisia dracunculus* var.*glauca* OR668915	*Artemisia dracunculus* var.*glauca* OR668915	*Artemisia dracunculus* var.*glauca* OR668915
9	*Artemisia dracunculus* ON685491	*Artemisia sieversiana* MG951499	*Berberis heteropoda* MT931850	*Artemisia sieversiana* MG951499	*Artemisia sieversiana* MG951499	*Artemisia vulgaris* KX581830	*Artemisia sieversiana* MG951499
10	*Artemisia vulgaris* KX58183 0	*Artemisia vulgaris* KX581932	*Betula chichibuensis* NC047177	*Artemisia vulgaris* OR464897	*Artemisia stolonifera* NC049572	*Aster poliothamnus* NC079626	*Artemisia stolonifera* NC049572
11	*Avena sativa* OV702300	*Aster souliei* NC073537	*Bistorta vivipara* ON229551	*Aster souliei* NC073537	*Aster souliei* NC073537	*Avena fatua* MN192753	*Aster souliei* NC073537
12	*Berberis heteropoda* MT923999	*Avena fatua* NC044170	*Bupleurum thianschanicum* OQ621996	*Avena fatua* NC044170	*Avena fatua* NC044170	*Berberis heteropoda* MT924001	*Avena fatua* NC044170
13	*Berberis heteropoda* MT924001	*Berberis thunbergii* NC067773	*Bupleurum triradiatum* OQ621997	*Berberis thunbergii* OR464917	*Berberis thunbergii* OR464917	*Berberis vulgaris* MW545987	*Berberis thunbergii* OR464917
14	*Betula nana* MN531381	*Betula papyrifera* KX677751	*Campanula glomerata* KM360690	*Betula ermanii* OR350411	*Betula ermanii* OR350411	*Betula pubescens* KX162899	*Betula chichibuensis* NC047177
15	*Bistorta vivipara* MT923436	*Bistorta vivipara* KX676568	*Campanula glomerata* MT930597	*Bistorta vivipara* ON229551	*Bistorta vivipara* ON229551	*Bistorta vivipara* JN235092	*Bistorta vivipara* MW417649
16	*Bupleurum sibiricum* OL473063	*Bupleurum rockii* OQ621992	*Campanula glomerata* MT930599	*Bupleurum thianschanicum* OQ621996	*Bupleurum thianschanicum* OQ621996	*Bupleurum smithii* OR493999	*Bupleurum thianschanicum* OQ621996
17	*Bupleurum thianschanicum* MT922582	*Bupleurum triradiatum* OQ621997	*Cannabis sativa* MH118118	*Bupleurum triradiatum* NC077624	*Bupleurum triradiatum* NC077624	*Bupleurum thianschanicum* MT922582	*Bupleurum triradiatum* NC077624
18	*Campanula glomerata* MK652011	*Campanula glomerata* JN894555	*Caragana jubata* MT211963	*Campanula glomerata* LT706563	*Campanula glomerata* LT706563	*Campanula glomerata* MT922852	*Campanula glomerata* LT706563
19	*Campanula glomerata* MT922852	*Campanula glomerata* KC146481	*Carpesium lipskyi* NC080873	*Campanula glomerata* subsp.*hispida* LR701366	*Campanula glomerata* subsp.*hispida* LR701370	*Campanula glomerata* MT922855	*Campanula glomerata* subsp.*hispida* LR701366
20	*Cannabis sativa* AB564722	*Campanula glomerata* LR701366	*Carum carvi* NC029889	*Campanula glomerata* var.*dahurica* KC146481	*Campanula glomerata* var.*dahurica* KC146481	*Campanula glomerata* MT930599	*Campanula glomerata* var.*dahurica* KC146481
21	*Carpesium triste* MH674412	*Cannabis sativa* KR184827	*Cerastium cerastoides* MT930978	*Cannabis sativa* HQ619806	*Cannabis sativa* HQ619806	*Cannabis sativa* OR642791	*Cannabis sativa* HQ619806
22	*Carum carvi* MH645765	*Caragana jubata* MT211963	*Cerastium cerastoides* OQ326798	*Caragana jubata* MT211963	*Caragana jubata* MT211963	*Caragana jubata* MK945647	*Caragana jubata* MT211963
23	*Cerastium cerastoides* MH219813	*Carpesium lipskyi* NC080873	*Clematis glauca* MW246166	*Carpesium lipskyi* NC080873	*Carpesium lipskyi* NC080873	*Carpesium triste* MH674412	*Carpesium lipskyi* NC080873
24	*Cerastium cerastoides* MH47794 0	*Carum carvi* MN167187	*Clematis tangutica* MK253446	*Carum carvi* KX344524	*Carum carvi* KX344524	*Carum carvi* MT359946	*Carum carvi* KX344524
25	*Clematis intricata* JN8 09683	*Centaurea cyanus* MK519886	*Conium Maculatum* KT178116	*Centaurea stoebe* OQ413806	*Centaurea montana* MG225131	*Centaurea procurrens* KY293573	*Centaurea stoebe* OQ413806
26	*Clematis tangutica* AB12 0195	*Centaurea stoebe* OQ413806	*Crataegus Monogyna* NC066002	*Cerastium cerastoides* MK926075	*Centaurea stoebe* OQ413806	*Cerastium cerastoides* MH477940	*Cerastium cerastoides* MK926075
27	*Codonopsis clematidea* KX645634	*Cerastium cerastoides* MK926075	*Cynoglossum amabile* NC061706	*Clematis glauca* MW246166	*Cerastium cerastoides* MK926075	*Cerastium cerastoides* OQ324759	*Clematis glauca* MW246166
28	*Conium Maculatum* MZ191 027	*Clematis glauca* ON520701	*Dasiphora fruticosa* MG735728	*Clematis tangutica* MK253446	*Clematis glauca* MW246166	*Clematis glauca* MW246166	*Clematis tangutica* MK253446
29	*Cynoglossum officinale* KU927696	*Clematis tangutica* MK253446	*Daucus carota* KX832308	*Codonopsis clematidea* KT970202	*Clematis tangutica* MK253446	*Clematis tangutica* AB120195	*Codonopsis clematidea* KT970202
30	*Daucus carota* MH711237	*Codonopsis clematidea* KT970202	*Dictamnus dasycarpus* NC063821	*Conium maculatum* KX676957	*Codonopsis clematidea* KT970202	*Codonopsis clematidea* KX645634	*Conium maculatum* KX676957
31	*Dictamnus angustifolius* MT924084	*Conium Maculatum* KX676957	*Doronicum altaicum* MN185073	*Crataegus songarica* OR915930	*Conium maculatum* KX676957	*Conium maculatum* MZ191027	*Crataegus monogyna* MF350263
32	*Doronicum altaicum* MT922693	*Crataegus Monogyna* MF350263	*Dracocephalum Moldavica* NC062584	*Cynoglossum officinale* HQ593260	*Crataegus songarica* OR915930	*Cynoglossum officinale* KU927696	*Cynoglossum officinale* HQ593260
33	*Dracocephalum grandiflorum* OR197143	*Cynoglossum amabile* NC061706	*Dracocephalum palmatum* NC031874	*Dasiphora fruticosa* KX677540	*Cynoglossum officinale* HQ593260	*Dasiphora fruticosa* MZ366409	*Dasiphora fruticosa* KX677540
34	*Dracocephalum integrifolium* MT923746	*Cynoglossum officinale* EU599664	*Dracocephalum taliense* NC058336	*Daucus carota* subsp.*maximus* KX832308	*Dasiphora fruticosa* KX677540	*Daucus carota* subsp.*sativus* CP093348	*Daucus carota subsp.maximus* KX832308
35	*Dracocephalum nutans* KF041157	*Dasiphora fruticosa* MK925837	*Epilobium ulleungensis* NC039575	*Dictamnus dasycarpus* MZ677241	*Daucus carota* subsp.*maximus* KX832308	*Dictamnus dasycarpus* NC063821	*Dictamnus dasycarpus* MZ677241
36	*Epilobium palustre* MH711656	*Daucus carota* KX832308	*Epipactis helleborine* NC041185	*Doronicum altaicum* MT922693	*Dictamnus dasycarpus* MZ677241	*Doronicum altaicum* MT922693	*Doronicum altaicum* MT922693
37	*Epipactis helleborine* KU931728	*Dictamnus dasycarpus* MZ677241	*Erigeron canadensis* NC046789	*Dracocephalum grandiflorum* MT929814	*Doronicum altaicum* MT917438	*Dracocephalum grandiflorum* OR197143	*Dracocephalum grandiflorum* MT929814
38	*Erigeron canadensis* MH7681 05	*Doronicum altaicum* MK435704	*Erysimum flavum* NC081120	*Dracocephalum integrifolium* MW924787	*Dracocephalum grandiflorum* MT929814	*Dracocephalum integrifolium* MT923746	*Dracocephalum integrifolium* MW924787
39	*Erigeron lachnocephalus* MT922713	*Dracocephalum grandiflorum* MT929814	*Erysimum inconspicuum* JX848441	*Epilobium montanum* MK925723	*Dracocephalum integrifolium* MW924787	*Dracocephalum moldavica* MT931585	*Epilobium montanum* MK925723
4 0	*Erysimum flavum* MT923054	*Dracocephalum integrifolium* MW924787	*Erysimum siliculosum* MT930941	*Epipactis helleborine* NC041185	*Epilobium palustre* MK520058	*Epilobium palustre* MT784096	*Epipactis helleborine* HQ593283
41	*Erysimum incanum* KF849864	*Dracocephalum Moldavica* MN311836	*Gagea serotina* MT931703	*Epipremnum aureum* MK286107	*Epipactis helleborine* HQ593283	*Epipactis helleborine* KU931728	*Epipremnum aureum* MK286107

42	*Erysimum kotuchovii* OP558070	*Epilobium palustre* MH660210	*Glycyrrhiza uralensis* MT120791	*Erigeron canadensis* NC046789	*Epipremnum aureum* MK286107	*Erigeron canadensis* NC046789	*Erigeron canadensis* NC046789
43	*Gagea serotina* LN874806	*Epipactis helleborine* NC041185	*Glycyrrhiza uralensis* MT931422	*Erysimum cheiranthoides* JN893990	*Erigeron canadensis* NC046789	*Erysimum flavum* MT923054	*Erysimum cheiranthoides* JN893990
44	*Glycyrrhiza inflata* KY860932	*Epipremnum pinnatum* GU135036	*Heracleum yungningense* NC047287	*Erysimum flavum* NC081120	*Erysimum cheiranthoides* JN893990	*Erysimum flavum* NC081120	*Erysimum flavum* NC081120
45	*Glycyrrhiza uralensis* KM588200	*Erigeron canadensis* NC046789	*Humulus lupulus* MG573060	*Erysimum teretifolium* MN626598	*Erysimum flavum* NC081120	*Erysimum flavum* subsp.*altaicum* OQ644460	*Erysimum teretifolium* MN626598
46	*Heracleum dissectum* OL473019	*Erysimum cheiranthoides* JN893989	*Hyoscyamus niger* NC024261	*Gagea serotina* KM085627	*Erysimum odoratum* NC049638	*Gagea serotina* MT931703	*Gagea serotina* KM085627
47	*Humulus lupulus* MH712704	*Erysimum flavum* NC081120	*Inula japonica* NC063729	*Glycyrrhiza uralensis* MT120790	*Gagea serotina* KM085627	*Glycyrrhiza uralensis* KY860930	*Glycyrrhiza uralensis* MT120791
48	*Hyoscyamus niger* MH809159	*Gagea serotina* MK926005	*Inula racemosa* MN185077	*Glycyrrhiza uralensis* MT120791	*Glycyrrhiza uralensis* MT120791	*Glycyrrhiza uralensis* ON009073	*Heracleum moellendorffii* MH659043
49	*Inula racemosa* MH808140	*Glycyrrhiza uralensis* MT120791	*Inula rhizocephala* MT930467	*Heracleum moellendorffii* MH659043	*Heracleum moellendorffii* MH659043	*Heracleum dissectum* OL473019	*Humulus lupulus* OR464988
50	*Inula rhizocephala* MT922730	*Glycyrrhiza uralensis* OQ722366	*Iris anguifuga* KP089552	*Humulus lupulus* OR464988	*Humulus lupulus* OR464988	*Humulus lupulus* MH712704	*Hyoscyamus niger* NC024261
51	*Inula salicina* KT119553	*Heracleum Moellendorffii* MH659043	*Juniperus sabina* NC039644	*Hyoscyamus niger* NC024261	*Hyoscyamus niger* NC024261	*Hyoscyamus niger* MH809159	*Inula ensifolia* KY397491
52	*Juniperus oxycedrus* LC42 0974	*Humulus lupulus* MG573060	*Lamium album* NC036971	*Inula ensifolia* KY397491	*Inula racemosa* MK435714	*Inula racemosa* MH808140	*Inula racemosa* MK435714
53	*Lamium album* OK166643	*Hyoscyamus niger* NC024261	*Lathyrus pratensis* MF158762	*Inula racemosa* MK435714	*Inula rhizocephala* MT917469	*Inula rhizocephala* MT922730	*Inula rhizocephala* MT917469
54	*Lathyrus pratensis* LS973913	*Inula japonica* HM989789	*Lathyrus tuberosus* MT931441	*Inula rhizocephala* MT917469	*Juniperus pseudosabina* NC077615	*Inula salicina* KT119553	*Juniperus pseudosabina* NC077615
55	*Lathyrus tuberosus* MT923614	*Inula racemosa* MK435714	*Leontopodium leontopodioides* GQ436452	*Juniperus pseudosabina* NC077615	*Lamium album* MN204957	*Juniperus sabina* OR505052	*Lamium album subsp.album* OK166643
56	*Leontopodium leontopodioides* MT922782	*Juniperus pseudosabina* OQ644238	*Leontopodium leontopodioides* NC072092	*Lamium album subsp.album* OK166643	*Lathyrus pratensis* KX677550	*Lamium album* subsp.*album* OK166643	*Lathyrus pratensis* KX677550
57	*Leontopodium leontopodioides* OP950266	*Lamium album* MT929859	*Leonurus turkestanicus* MT931599	*Lathyrus pratensis* KX677550	*Lathyrus tuberosus* MT918290	*Lathyrus pratensis* MF158762	*Lathyrus tuberosus* MT923614
58	*Leonurus turkestanicus* EF395809	*Lathyrus pratensis* KX677550	*Ligularia alpigena* MT930523	*Lathyrus tuberosus* MT923614	*Leontopodium leontopodinum* GU943484	*Lathyrus tuberosus* MT923614	*Leontopodium leontopodinum* GU943484
59	*Libanotis schrenkiana* LC195541	*Lathyrus tuberosus* MT918290	*Lonicera hispida* NC040962	*Leontopodium leontopodinum* GU943484	*Leontopodium leontopodioides* NC072092	*Leontopodium leontopodioides* MT922782	*Leontopodium leontopodioides* NC072092
60	*Ligularia alpigena* MT922784	*Leontopodium leontopodinum* GU943484	*Malva sylvestris* MK521565	*Leontopodium leontopodioides* NC072092	*Leonurus turkestanicus* EF395812	*Leontopodium leontopodioides* OP950266	*Leonurus turkestanicus* EF395812
61	*Ligularia heterophylla* MT922785	*Leontopodium leontopodioides* NC072092	*Matricaria Matricarioides* MN204756	*Leonurus turkestanicus* EF395812	*Ligularia intermedia* MK435728	*Leonurus turkestanicus* EF395809	*Ligularia intermedia* MK435728
62	*Lonicera altmannii* EF462216	*Leonurus cardiaca* MZ274170	*Medicago lupulina* MN601454	*Ligularia intermedia* MK435728	*Ligularia veitchiana* NC039385	*Libanotis seseloides* OL472944	*Ligularia veitchiana* NC039385
63	*Malva pusilla* KJ999382	*Ligularia hodgsonii* MK435724	*Mentha spicata* NC037247	*Ligularia veitchiana* NC039385	*Lonicera hispida* NC040962	*Ligularia alpigena* MT922784	*Lonicera hispida* NC040962
64	*Matricaria matricarioides* MH537768	*Ligularia veitchiana* NC039385	*Myricaria bracteata* AY099907	*Lonicera hispida* NC040962	*Malva neglecta* EU346788	*Lonicera elisae* MH936541	*Malva neglecta* EU346788
65	*Medicago lupulina* MT610942	*Lonicera hispida* NC040962	*Orthilia secunda* AF419838	*Malva neglecta* EU346788	*Malva pusilla* MH659693	*Malva pusilla* KJ999382	*Malva pusilla* MH659693
66	*Mentha sp.* MW915670	*Malva neglecta* JN894571	*Oxyria digyna* MN204823	*Malva pusilla* MH659693	*Matricaria occidentalis* MF963679	*Matricaria matricarioides* MH537768	*Matricaria discoidea* HM850671
67	*Myricaria bracteata* MT923440	*Malva pseudolavatera* MH078525	*Oxytropis globiflora* MT931465	*Matricaria occidentalis* MF963679	*Medicago lupulina* KJ204508	*Medicago lupulina* MT610942	*Medicago lupulina* MT610942
68	*Orthilia secunda* KR005149	*Matricaria occidentalis* MF963679	*Papaver nudicaule* ON209191	*Medicago lupulina* MT610942	*Mentha aquatica* var.*citrata* KX879092	*Mentha sp.* MW915670	*Mentha aquatica var.citrata* KX879092
69	*Oxyria digyna* KC691703	*Medicago lupulina* KJ204508	*Parnassia palustris* NC045280	*Mentha aquatica var.citrata* KX879092	*Myricaria squamosa* OP778409	*Myricaria bracteata* MT923440	*Myricaria bracteata* MT923440
70	*Oxytropis globiflora* MT923637	*Mentha longifolia* MF694865	*Pedicularis striata* OQ842970	*Myricaria bracteata* MT923440	*Orthilia secunda* JN894293	*Orthilia secunda* KR005149	*Oreomecon nudicaulis* ON209191
71	*Papaver nudicaule* MT924019	*Myricaria bracteata* MT918093	*Phleum pratense* NC067044	*Orthilia secunda* JN894293	*Oxyria digyna* KR003692	*Oxyria digyna* MT931206	*Orthilia secunda* JN894293
72	*Parnassia pusilla* MZ568374	*Orthilia secunda* JN894293	*Plantago Major* ON684496	*Oxyria digyna* KR003692	*Oxytropis arctobia* NC050861	*Oxytropis pilosa* KX954880	*Oxyria digyna* KR003692
73	*Phleum pratense* KU883519	*Oxyria digyna* KR003692	*Poa pratensis* JX848506	*Oxytropis podocarpa* KX676897	*Papaver hybridum* JN895926	*Papaver nudicaule* MT924019	*Oxytropis podocarpa* KX676897
74	*Plantago Major* MW139237	*Oxytropis podocarpa* KX676897	*Polemonium caeruleum* MT931300	*Papaver hybridum* JN896018	*Papaver hybridum* JN896018	*Parnassia bifolia* MT924116	*Papaver hybridum* JN896018
75	*Poa glauca* JN999391	*Papaver hybridum* JN895926	*Potentilla chinensis* MN871983	*Papaver nudicaule* ON209191	*Papaver nudicaule* ON209191	*Pedicularis physocalyx* MF158766	*Parnassia bifolia* MT930200
76	*Polemonium caeruleum* MT923485	*Papaver hybridum* JN896018	*Primula algida* MT931334	*Parnassia bifolia* MT930200	*Parnassia bifolia* MT930200	*Phleum pratense* MK050855	*Phleum pratense* MH888114
77	*Potentilla fruticosa* AF163478	*Papaver nudicaule* MN434010	*Primula algida* NC053582	*Phleum pratense* MH888114	*Phleum pratense* MH888114	*Plantago media* OK523417	*Plantago media* NC028520
78	*Potentilla reptans* FN430815	*Parnassia bifolia* MT930200	*Prunus armeniaca* MH700954	*Plantago media* NC028520	*Plantago media* NC028520	*Poa pratensis* JX848506	*Poa pratensis* NC057962
79	*Primula algida* MT923501	*Phleum pratense* MH888114	*Prunus padus* NC026982	*Poa pratensis* NC057962	*Poa pratensis* NC057962	*Polemonium caeruleum* MT923485	*Polemonium caeruleum* MT918164
80	*Primula algida* MT923502	*Plantago Major* JN407176	*Pyrola rotundifolia* KU833271	*Polemonium caeruleum* MT918164	*Polemonium caeruleum* MT918164	*Potentilla reptans* FN430815	*Potentilla reptans* JN895282
81	*Prunus armeniaca* MG735475	*Poa pratensis* NC057962	*Rheum tanguticum* MN564929	*Potentilla reptans* JN895282	*Potentilla reptans* JN895282	*Primula algida* MT923501	*Primula algida* MT918142
82	*Prunus padus* AF318726	*Polemonium caeruleum* MT918164	*Rhodiola kirilowii* MT460451	*Primula algida* MT918142	*Primula algida* MT918141	*Primula algida* MT923502	*Primula algida* MT923502
83	*Pyrola rotundifolia* MN443050	*Potentilla reptans* JN895282	*Rhodiola quadrifida* NC060680	*Primula algida* MT923502	*Primula algida* MT918142	*Prunus padus* AF318726	*Prunus armeniaca* MH700954
84	*Rhaponticoides ruthenica* MT922671	*Primula algida* MT918141	*Roemeria refracta* MF973016	*Prunus armeniaca* MH700954	*Prunus armeniaca* MH700954	*Prunus salicina var.cordata* OR726647	*Prunus padus* subsp.*borealis* KP769142
85	*Rheum wittrockii* KF258686	*Primula algida* MT918142	*Roemeria refracta* MF973017	*Prunus padus subsp.borealis* KP769142	*Prunus padus* subsp.*borealis* KP769142	*Pyrola rugosa* MH711293	*Pyrola rotundifolia* MT862711
86	*Rhodiola* kirilowii MT924099	*Prunus armeniaca* MH700954	*Rosa beggeriana* NC079961	*Pyrola rotundifolia* MT862711	*Pyrola rotundifolia* MT862711	*Rheum tanguticum* var.*liupanshanense* OR652288	*Rheum wittrockii* MT918089

87	Rhodiola *quadrifida* MT924103	*Prunus padus* KP769142	*Rubus idaeus* JX848533	*Rheum wittrockii* MT918089	*Rheum wittrockii* MT918089	*Rhodiola kirilowii* MT924099	*Rhodiola kirilowii* MT460451
88	*Roemeria refracta* DQ250299	*Pyrola rotundifolia* MT862711	*Rubus sachalinensis* NC056965	*Rhodiola kirilowii* MT460451	*Rhodiola kirilowii* MT460451	*Rhodiola quadrifida* MT924103	*Rhodiola quadrifida* OM161976
89	*Roemeria refracta* KM044472	*Rheum wittrockii* MT918089	*Salvia deserta* MT156378	*Rhodiola quadrifida* OM161976	*Rhodiola quadrifida* OM161976	*Roemeria refracta* KM044472	*Rosa beggeriana* NC079961
90	*Rosa laxa* MT924077	*Rhodiola kirilowii* MT460451	*Salvia japonica* NC035233	*Rosa beggeriana* NC079961	*Rosa beggeriana* NC079961	*Roemeria refracta* MF973017	*Rubus idaeus* KM036626
91	*Rubus allegheniensis* KF977442	*Rhodiola quadrifida* KP114858	*Saussurea superba* NC072929	*Rubus idaeus* KM036626	*Rubus idaeus* KM036626	*Rosa laxa* MT924077	*Rubus sachalinensis* NC056965
92	*Rubus idaeus* AF055757	*Rosa beggeriana* NC079961	*Saxifraga cernua* NC070450	*Rubus sachalinensis* NC056965	*Rubus sachalinensis* NC056965	*Rubus allegheniensis* KF977442	*Salvia deserta* JQ934055
93	*Salvia deserta* JQ934119	*Rubus idaeus* KM036626	*Sophora alopecuroides* NC045070	*Salvia deserta* JQ934055	*Salvia deserta* JQ934055	*Rubus idaeus* KM037405	*Saxifraga cernua* KC475826
94	*Saxifraga cernua* LN812384	*Rubus sachalinensis* NC056965	*Sorbus prattii* MK814479	*Saxifraga cernua* KC475826	*Saxifraga cernua* KC475826	*Salvia deserta* NC084045	*Sophora alopecuroides* MT923644
95	*Sophora alopecuroides* MT923644	*Salvia deserta* JQ934055	*Sorbus tianschanica* MK920289	*Sophora alopecuroides* MT923644	*Sophora alopecuroides* MN311792	*Saxifraga cernua* NC070450	*Sorbus cashmiriana* OR915991
96	*Sorbus tianschanica* MN215998	*Saxifraga cernua* MK926397	*Sparganium stoloniferum* NC044634	*Sorbus cashmiriana* OR915991	*Sorbus cashmiriana* OR915991	*Sophora alopecuroides* MT923644	*Sorbus tianschanica* MN215998
97	Sorbus *tianschanica* MT924081	*Sophora alopecuroides* NC045070	*Thalictrum simplex* NC068627	*Sorbus tianschanica* MN215998	*Sorbus epidendron* OR915990	*Sorbus tianschanica* MN215998	*Sparganium stoloniferum* NC044634
98	*Sparganium stoloniferum* KF265395	*Sorbus aucuparia* MK925731	*Thymus japonicus* NC046822	*Sparganium stoloniferum* NC044634	*Sparganium stoloniferum* NC044634	*Sorbus tianschanica* MT924081	*Thalictrum simplex* NC068627
99	*Thalictrum simplex* MT924037	*Sparganium stoloniferum* NC044634	*Thymus vulgaris* OP418352	*Thalictrum simplex* NC068627	*Thalictrum simplex* NC068627	*Sparganium stoloniferum* KF265395	*Thymus quinquecostatus* LC618903
100	*Thymus marschallianus* OP673522	*Thalictrum simplex* NC068627	*Triglochin palustris*DQ859176	*Thymus quinquecostatus* LC618903	*Thymus quinquecostatus* LC618903	*Thalictrum simplex* MT924037	*Thymus serpyllum* MK925827
101	*Thymus quinquecostatus* EU556524	*Thymus serpyllum* MK925827	*Trigonotis cavaleriei* OP787129	*Thymus serpyllum* MK925827	*Thymus serpyllum* MK925827	*Thymus diminutus* MZ366433	*Triglochin palustris* KF632820
102	*Triglochin palustris* KX165519	*Thymus striatus* HE819413	*Valeriana officinalis* MT931271	*Triglochin palustris* KF632820	*Triglochin palustris* KF632820	*Thymus sylvestris* ON685350	*Valeriana officinalis* JN896022
103	*Valeriana officinalis* EU796889	*Triglochin palustris* KF632820	*Verbascum thapsus* KT178130	*Valeriana officinalis* JN896022	*Valeriana officinalis* JN896022	*Triglochin palustris* KP174267	*Verbascum thapsus* OQ434020
104	*Verbascum thapsus* OL763335	*Valeriana officinalis* NC045052	*Veronica nakaiana* NC031153	*Verbascum thapsus* OQ434020	*Verbascum thapsus* OQ434020	*Valeriana officinalis* ON684518	*Veronica nakaiana* NC031153
105	*Veronica alatavica* KU047989	*Verbascum thapsus* OQ434020	*Vicia cracca* NC056383	*Veronica nakaiana* NC031153	*Veronica nakaiana* NC031153	*Verbascum thapsus* OY770119	*Vicia cracca* JN894329
106	*Vicia cracca* MT923665	*Veronica nakaiana* NC031153	*Xanthisma spinulosum* JX848434	*Vicia cracca* JN894329	*Vicia cracca* JN894329	*Veronica ferganica* MT923822	
107		*Vicia cracca* KX677490				*Vicia cracca* MT923665	

Table S3 Identification success rates at species level, generic level, and family level using BLAST(%)

Different Level	ITS	*matK*	*rbcL*	ITS+*matK*+*rbcL*	ITS+*matK*	ITS+*rbcL*	*matK*+*rbcL*
species-level resolution	71.29	60.40	74.65	65.35	65.35	65.35	61.39
genus-level resolution	92.66	90.74	94.55	92.38	92.66	92.52	91.59
family-level resolution	93.58	95.41	96.36	98.1	97.25	96.26	97.2

Table S4 Pairwise identification for each locus and multi-loci markers by BLAST in this study

Species Name	Pairwise Identify (ITS)	Pairwise Identify (*matK*)	Pairwise Identify (*rbcL*)	Pairwise Identify (ITS+*matK*)	Pairwise Identify (matK+*rbcL*)	Pairwise Identify (ITS+*rbcL*)	Pairwise Identify (ITS+*matK* +*rbcL*)
Aconitum leucostomum	99.90	99.90	99.30	99.90	0.00	0.00	0.00
Aconitum monticola	0.00	0.00	100.00	0.00	0.00	99.50	0.00
Amaranthus retroflexus	100.00	99.80	99.70	99.90	99.90	100.00	99.90
Androsace septentrionalis	100.00	99.90	100.00	99.90	99.90	100.00	99.90
Aquilegia atrovinosa	0	0.00	0.00	0.00	0.00	0.00	0.00
Arctium tomentosum	100.00	99.70	0.00	99.70	99.70	100.00	99.70
Arnebia euchroma	99.80	99.30	100.00	99.80	99.80	100.00	99.80
Artemisia dracunculus	100.00	99.80	99.30	100.00	100.00	100.00	100.00
Artemisia tanacetifolia	0.00	0.00	0.00	0.00	0.00	0.00	0.00
Aster alpinus	0.00	0.00	0.00	0.00	0.00	0.00	0.00
Avena fatua	0.00	99.90	99.80	99.90	99.90	99.80	99.90
Berberis iliensis	0.00	0.00	0.00	0.00	0.00	0.00	0.00
Betula tianschanica	0.00	0.00	0.00	0.00	0.00	0.00	0.00
Bistorta vivipara	100.00	99.70	99.30	99.70	99.70	99.90	99.90
Bupleurum thianschanicum	0.00	0.00	0.00	99.70	0.00	0.00	99.70
Bupleurum triradiatum	0.00	99.90	99.50	99.90	99.90	0.00	99.90
Campanula glomerata	99.70	99.90	99.80	99.90	100.00	99.40	99.90
Campanula glomerata	100.00	99.90	100.00	100.00	99.90	100.00	100.00
Campanula glomerata subsp.*speciosa*	99.80	99.90	100.00	99.90	99.90	99.80	99.90
Cannabis sativa	100.00	99.60	100.00	100.00	100.00	100.00	100.00
Caragana jubata	99.46	99.70	99.30	99.70	99.70	99.50	99.70
Carpesium triste	99.90	0.00	0.00	0.00	0.00	99.90	0.00
Carum carvi	100.00	99.80	99.70	99.90	99.90	100.00	99.90
Clematis glauca	0.00	99.50	99.50	99.90	99.90	100.00	99.90
Clematis tangutica	100.00	100.00	99.80	100.00	100.00	100.00	100.00
Codonopsis clematidea	100.00	99.50	0.00	99.90	99.90	100.00	99.90
Conium maculatum	100.00	100.00	99.50	100.00	99.90	100.00	99.90
Crataegus songarica	0.00	0.00	0.00	100.00	100.00	0.00	0.00
Cynoglossum divaricatum	0.00	0.00	0.00	0.00	0.00	0.00	0.00
Cynoglossum officinale	100.00	0.00	99.80	99.90	99.90	100.00	99.88
Dasiphora fruticosa	99.80	0.00	100.00	99.90	99.90	99.60	99.90
Daucus carota	100.00	99.20	100.00	100.00	100.00	99.90	100.00
Dichodon cerastoides	100.00	0.00	99.80	0.00	100.00	99.90	0.00
Dichodon cerastoides	99.90	0.00	100.00	100.00	0.00	100.00	100.00
Dictamnus dasycarpus	0.00	99.90	99.50	99.90	99.90	99.80	99.90
Doronicum altaicum	100.00	99.70	100.00	100.00	99.90	100.00	100.00

Dracocephalum grandiflorum	99.70	99.30	99.50	99.30	99.30	100.00	99.30
Dracocephalum nutans	100.00	0.00	0.00	0.00	0.00	0.00	0.00
Dracocephalum ruyschiana	0.00	0.00	99.50	0.00	0.00	0.00	0.00
Elsholtzia densa	0.00	0.00	0.00	0.00	0.00	0.00	0.00
Epilobium palustre	99.00	99.60	100.00	0.00	99.60	99.10	0.00
Epipactis helleborine	100.00	99.70	99.50	99.90	99.90	100.00	99.90
Erigeron canadensis	100.00	99.50	100.00	99.90	99.90	100.00	99.90
Erysimum diffusum	0.00	0.00	0.00	0.00	0.00	0.00	0.00
Erysimum diffusum	0.00	0.00	0.00	0.00	0.00	0.00	0.00
Erysimum flavum subsp.*altaicum*	99.90	99.90	100.00	99.90	99.90	0.00	99.90
Gagea serotina	99.80	99.90	100.00	100.00	100.00	100.00	100.00
Glycyrrhiza uralensis	0.00	99.30	99.20	99.90	99.90	99.20	99.90
Glycyrrhiza uralensis	100.00	100.00	100.00	100.00	100.00	100.00	99.49
Heracleum dissectum	100.00	0.00	0.00	0.00	0.00	100.00	0.00
Humulus lupulus	100.00	99.50	100.00	100.00	100.00	100.00	100.00
Hyoscyamus niger	100.00	99.90	100.00	100.00	100.00	100.00	100.00
Inula britannica	0.00	0.00	0.00	0.00	0.00	0.00	0.00
Inula racemosa	100.00	99.60	100.00	99.70	99.70	100.00	99.70
Inula rhizocephala	99.70	0.00	99.80	99.70	99.70	99.90	99.70
Juniperus sabina	0.00	0.00	100.00	0.00	0.00	100.00	0.00
Lamium album	98.70	82.80	100.00	100.00	100.00	100.00	100.00
Lathyrus pratensis	100.00	99.90	100.00	100.00	100.00	100.00	100.00
Lathyrus tuberosus	100.00	100.00	100.00	100.00	100.00	100.00	100.00
Leontopodium leontopodioides	100.00	99.90	99.70	99.90	99.90	99.50	99.90
Leontopodium leontopodioides	99.50	99.90	100.00	99.90	99.90	99.70	0.00
Leonurus turkestanicus	98.80	0.00	99.50	99.50	99.50	98.80	99.50
Ligularia alpigena	100.00	0.00	0.00	0.00	0.00	100.00	0.00
Ligularia heterophylla	100.00	0.00	0.00	0.00	0.00	0.00	0.00
Lonicera hispida	0.00	99.10	99.10	99.40	99.40	0.00	99.40
Malva pusilla	99.90	0.00	100.00	100.00	0.00	0.00	100.00
Matricaria matricarioides	100.00	0.00	100.00	0.00	0.00	100.00	0.00
Medicago lupulina	100.00	99.90	99.80	100.00	99.90	100.00	100.00
Myricaria bracteata	100.00	100.00	100.00	100.00	0.00	100.00	100.00
Oreomecon nudicaulis	99.30	99.90	100.00	99.90	99.90	0.00	99.90
Origanum vulgare	0.00	0.00	0.00	0.00	0.00	0.00	0.00
Orthilia secunda	98.30	99.50	100.00	99.50	99.50	98.30	99.50
Oxyria digyna	99.40	99.50	100.00	99.90	99.90	100.00	99.90
Oxytropis ochroleuca	0.00	0.00	0.00	0.00	0.00	0.00	0.00
Parnassia palustris	0.00	0.00	99.00	0.00	0.00	0.00	0.00
Phleum pratense	99.90	99.60	99.80	99.70	99.70	99.90	99.70
Plantago media	97.20	99.20	99.50	0.00	0.00	0.00	99.90
Poa pratensis	0.00	99.20	99.80	99.40	99.40	99.80	99.40
Polemonium caeruleum	100.00	100.00	100.00	100.00	100.00	100.00	100.00
Potentilla chinensis	0.00	0.00	99.00	0.00	0.00	0.00	0.00
Primula algida	100.00	100.00	100.00	100.00	100.00	99.90	100.00
Primula algida	99.90	99.60	100.00	99.90	99.60	100.00	99.90
Prunus armeniaca	99.50	99.90	100.00	100.00	100.00	0.00	100.00
Prunus padus	99.90	100.00	100.00	100.00	100.00	99.90	100.00
Pseudolysimachion alatavicum	99.50	0.00	0.00	0.00	0.00	0.00	0.00

Pyrola rotundifolia	100.00	99.30	100.00	99.70	99.70	0.00	99.70
Rhaponticoides ruthenica	98.90	99.30	0.00	0.00	0.00	0.00	0.00
Rheum wittrockii	98.70	99.90	100.00	99.90	99.90	0.00	99.90
Rhodiola kirilowii	100.00	99.00	99.80	99.00	99.00	100.00	99.00
Rhodiola quadrifida	99.90	99.20	100.00	99.10	99.10	99.90	99.10
Roemeria refracta	100.00	0.00	100.00	0.00	0.00	100.00	100.00
Roemeria refracta	99.10	0.00	100.00	0.00	0.00	100.00	100.00
Rosa laxa	99.30	0.00	0.00	0.00	0.00	99.30	0.00
Rubus idaeus	99.90	99.90	99.70	99.90	99.90	99.70	99.90
Rubus sachalinensis	0.00	99.60	99.80	99.60	99.60	0.00	99.60
Salvia deserta	98.80	99.60	100.00	99.60	99.60	100.00	99.60
Salvia japonica	0.00	0.00	100.00	0.00	0.00	0.00	0.00
Saxifraga cernua	99.30	99.10	100.00	99.60	99.60	100.00	99.60
Sophora alopecuroides	100.00	99.70	99	100.00	99.90	100.00	100.00
Sorbus tianschanica	99.80	0.00	99.80	0.00	0.00	100.00	99.60
Sorbus tianschanica	99.90	0.00	100.00	99.60	0.00	99.80	0.00
Sparganium stoloniferum	100.00	99.60	100.00	99.60	99.60	100.00	99.60
Thalictrum simplex	99.90	99.90	100.00	99.90	99.90	99.90	99.90
Thymus marschallianus	99.90	0.00	0.00	0.00	0.00	0.00	0.00
Thymus proximus	0.00	0.00	0.00	0.00	0.00	0.00	0.00
Triglochin palustris	100.00	99.40	100.00	99.70	99.70	99.70	99.70
Valeriana officinalis	99.70	99.60	100.00	99.90	99.90	99.80	99.90
Verbascum thapsus	99.90	99.90	100.00	99.90	99.90	99.90	99.90
Vicia cracca	100.00	99.70	99.70	99.90	99.70	100	99.70

Notes:Pairwise Identification of 0 means that the best BLAST hit of the query sequence is not from the expected species.

Table S5 Species discrimination rate based on ABGD and ASAP for 109 samples of medicinal plants

Different Method	ITS	*matK*	*rbcL*	ITS+*matK*	ITS+*rbcL*	*matK*+*rbcL*	ITS+*MatK*+*rbcL*
ABGD	88.07() (96/109)	59.63% (65/109)	82.57% (90/109)	94.50% (103/109)	91.74% (100/109)	38.53% (42/109)	82.57% (90/109)
ASAP	77.06% (84/109)	94.50% (103/109)	88.99% (97/109)	85.32% (93/109)	81.65% (89/109)	88.99% (97/109)	88.99% (97/109)

Table S6 Identification success rates achieved by ML tree, Blast analysis method, ABGD method and ASAP method for each individual candidate barcode and their combinations.

Single candidate barcodes and combinations of them	ML tree Method	Similarity-based Method	Automatic Barcode Gap Discovery (ABGD)	Assemble Species by Automatic Partitioning (ASAP)
ITS	70.30%(71/101)	71.29%(72/101)	88.07%(96/109)	77.06%(84/109)
matk	62.38%(63/101)	60.40%(61/101)	59.63%(65/109)	94.50%(103/109)
rbcL	59.41%(60/101)	74.65%(75/101)	82.57%(90/109)	88.99%(97/109)
ITS +*matK*	32.67(33/101)	65.35%(66/101)	94.50%(103/109)	85.32%(93/109)
ITS+*rbcL*	38.61%(39/101)	65.35(66/101)	91.74%(100/109)	81.65%(89/109)
matK+*rbcL*	39.60%(40/101)	61.39(62/101)	38.53%(42/109)	88.99%(97/109)
ITS+*matK*+*rbcL*	26.73%(27/101)	65.35(66/101)	82.57%(90/109)	88.99%(97/109)

Notes:The species discriminating ability of the ML evolutionary tree was calculated based on the monophyletic analysis with conspecific formation in the formed phylogenetic tree (the discriminating power of the ML evolutionary tree = the number of species clustered branches with same species/the total number of species in this study).

Table S7. The evaluation value

Evaluation Value	Level	Description of status
0.6～1	I	When a biological genetic resource has a small amount of reserves or high utilisation value, the biological genetic resource is classified as level I.
0.5～0.6	II	When a biological genetic resource does not reach Grade 1 standard, but has a small amount of reserves or high utilisation value, the biological genetic resource is classified as level II.
0～0.4	III	When a biological genetic resource does not reach the standard of level III, the biological genetic resource is classified as general biological genetic resource.

Table S8 101 species ranked by hierarchical analysis of hierarchy (AHP)

Scientific Name	Family	IUCN Red List	AHP Value
Leontopodium leontopodioides	Asteraceae	LC	0.28
Clematis tangutica	Ranunculaceae	LC	0.31
Dichodon cerastoides	Caryophyllaceae	LC	0.32
Erysimum diffusum	Brassicaceae	-	0.33
Plantago media	Plantaginaceae	LC	0.38
Dracocephalum grandiflorum	Lamiaceae	LC	0.38
Crataegus songarica	Rosaceae	LC	0.39
Ligularia alpigena	Asteraceae	LC	0.39
Rhaponticoides ruthenica	Asteraceae	LC	0.39
Berberis iliensis	Berberidaceae	LC	0.40
Amaranthus retroflexus	Amaranthaceae	-	0.51
Clematis glauca	Ranunculaceae	LC	0.52
Bupleurum triradiatum	Apiaceae	LC	0.53
Caragana jubata	Fabaceae	LC	0.53
Dasiphora fruticosa	Rosaceae	-	0.53
Juniperus sabina	Cupressaceae	LC	0.53
Sorbus tianschanica	Rosaceae	LC	0.54
Prunus padus	Rosaceae	LC	0.54
Salvia deserta	Lamiaceae	LC	0.54
Avena fatua	Poaceae	-	0.55
Erigeron canadensis	Asteraceae	-	0.55
Salvia japonica	Lamiaceae	LC	0.55
Phleum pratense	Poaceae	LC	0.56
Elsholtzia densa	Lamiaceae	LC	0.56
Lamium album	Lamiaceae	LC	0.56
Lathyrus pratensis	Fabaceae	LC	0.56
Medicago lupulina	Fabaceae	LC	0.56
Sophora alopecuroides	Fabaceae	LC	0.56
Poa pratensis	Poaceae	LC	0.56
Saxifraga cernua	Saxifragaceae	LC	0.56
Polemonium caeruleum	Polemoniaceae	LC	0.56
Malva pusilla	Malvaceae	LC	0.56

Campanula glomerata	Campanulaceae	LC	0.56
Campanula glomerata subsp.*speciosa*	Campanulaceae	LC	0.56
Carpesium triste	Asteraceae	LC	0.56
Inula britannica	Asteraceae	LC	0.56
Aster alpinus	Asteraceae	LC	0.56
Epipactis helleborine	Orchidaceae	LC	0.56
Bistorta vivipara	Polygonaceae	-	0.56
Valeriana officinalis	Caprifoliaceae	LC	0.56
Parnassia palustris	Celastraceae	LC	0.56
Sparganium stoloniferum	Typhaceae	LC	0.56
Verbascum thapsus	Scrophulariaceae	LC	0.56
Cynoglossum divaricatum	Boraginaceae	LC	0.56
Orthilia secunda	Ericaceae	LC	0.56
Oxyria digyna	Polygonaceae	LC	0.56
Hyoscyamus niger	Solanaceae	LC	0.56
Origanum vulgare	Lamiaceae	LC	0.56
Oreomecon nudicaulis	Papaveraceae	-	0.56
Dictamnus dasycarpus	Rutaceae	LC	0.56
Daucus carota	Apiaceae	-	0.56
Vicia cracca	Fabaceae	LC	0.56
Artemisia tanacetifolia	Asteraceae	LC	0.56
Epilobium palustre	Onagraceae	LC	0.56
Thalictrum simplex	Ranunculaceae	LC	0.56
Potentilla chinensis	Rosaceae	LC	0.56
Carum carvi	Apiaceae	LC	0.56
Triglochin palustris	Juncaginaceae	LC	0.56
Arnebia euchroma	Boraginaceae	EN	0.56
Myricaria bracteata	Tamaricaceae	LC	0.56
Pyrola rotundifolia	Ericaceae	LC	0.56
Rubus sachalinensis	Rosaceae	-	0.56
Rubus idaeus	Rosaceae	LC	0.56
Lonicera hispida	Caprifoliaceae	LC	0.57
Rhodiola kirilowii	Crassulaceae	LC	0.57
Artemisia dracunculus	Asteraceae	-	0.57
Bupleurum thianschanicum	Apiaceae	LC	0.57
Androsace septentrionalis	Primulaceae	LC	0.58
Matricaria matricarioides	Asteraceae	LC	0.58
Dracocephalum ruyschiana	Lamiaceae	LC	0.59
Dracocephalum nutans	Lamiaceae	LC	0.59
Codonopsis clematidea	Campanulaceae	LC	0.59
Inula rhizocephala	Asteraceae	DD	0.59
Aconitum leucostomum	Ranunculaceae	DD	0.59
Heracleum dissectum	Apiaceae	LC	0.59
Erysimum flavum subsp.*altaicum*	Brassicaceae	LC	0.59
Humulus lupulus	Cannabaceae	LC	0.59
Cannabis sativa	Cannabaceae	LC	0.38
Gagea serotina	Liliaceae	-	0.60
Primula algida	Primulaceae	LC	0.60
Leonurus turkestanicus	Lamiaceae	LC	0.60

Lathyrus tuberosus	Fabaceae	LC	0.60
Inula racemosa	Asteraceae	LC	0.60
Ligularia heterophylla	Asteraceae	LC	0.60
Rheum wittrockii	Polygonaceae	LC	0.60
Aquilegia atrovinosa	Ranunculaceae	LC	0.60
Cynoglossum officinale	Boraginaceae	-	0.60
Doronicum altaicum	Asteraceae	LC	0.60
Arctium tomentosum	Asteraceae	LC	0.60
Conium maculatum	Apiaceae	LC	0.61
Oxytropis ochroleuca	Fabaceae	LC	0.61
Roemeria refracta	Papaveraceae	LC	0.61
Thymus marschallianus	Lamiaceae	LC	0.61
Thymus proximus	Lamiaceae	LC	0.61
Rosa laxa	Rosaceae	LC	0.62
Prunus armeniaca	Rosaceae	NT	0.63
Betula tianschanica	Betulaceae	LC	0.63
Glycyrrhiza uralensis	Fabaceae	NT	0.65
Rhodiola quadrifida	Crassulaceae	NT	0.65
Pseudolysimachion alatavicum	Plantaginaceae	NT	0.69
Aconitum monticola	Ranunculaceae	NT	0.69

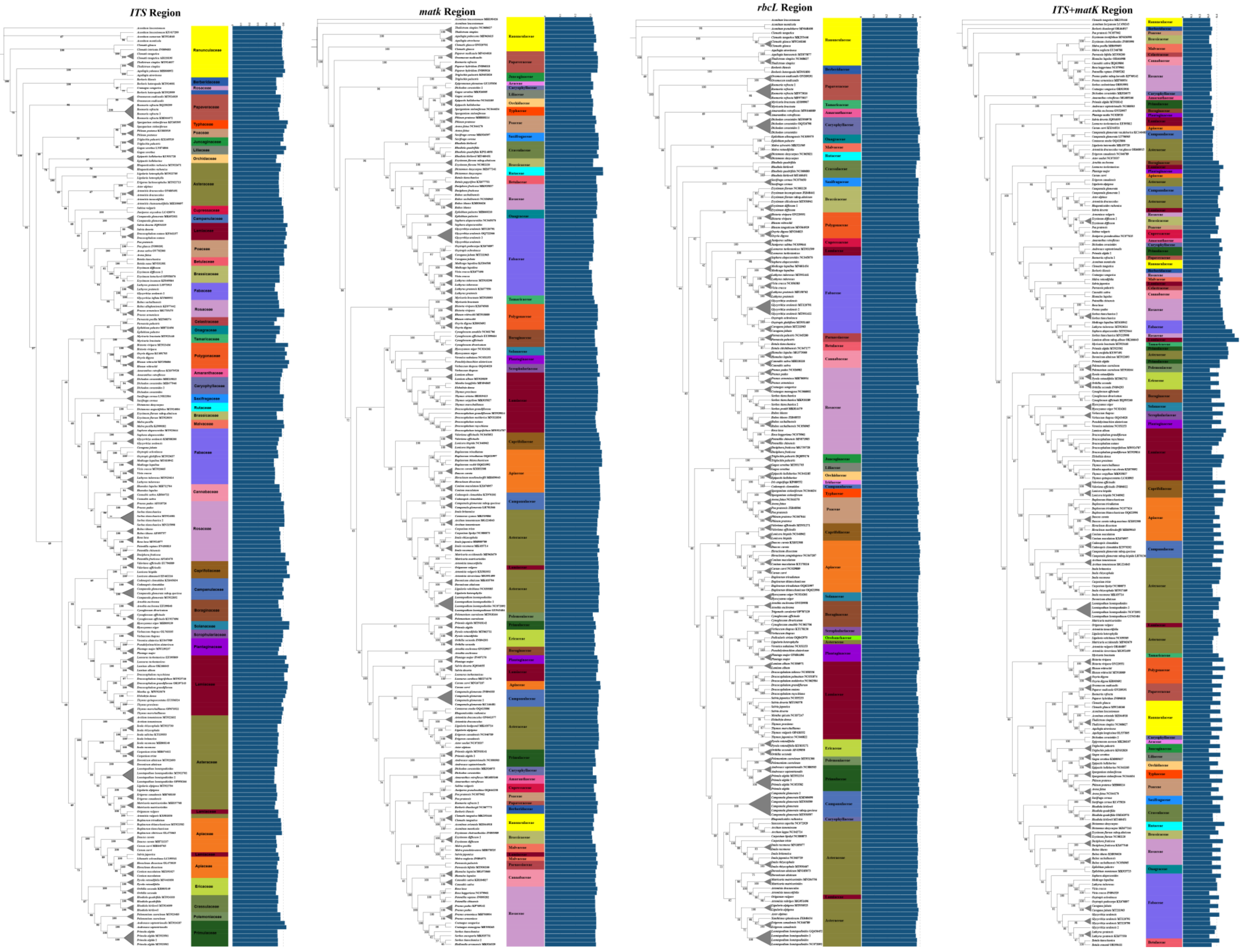
ITS Region
matk Region
rbcL Region
ITS+matK Region

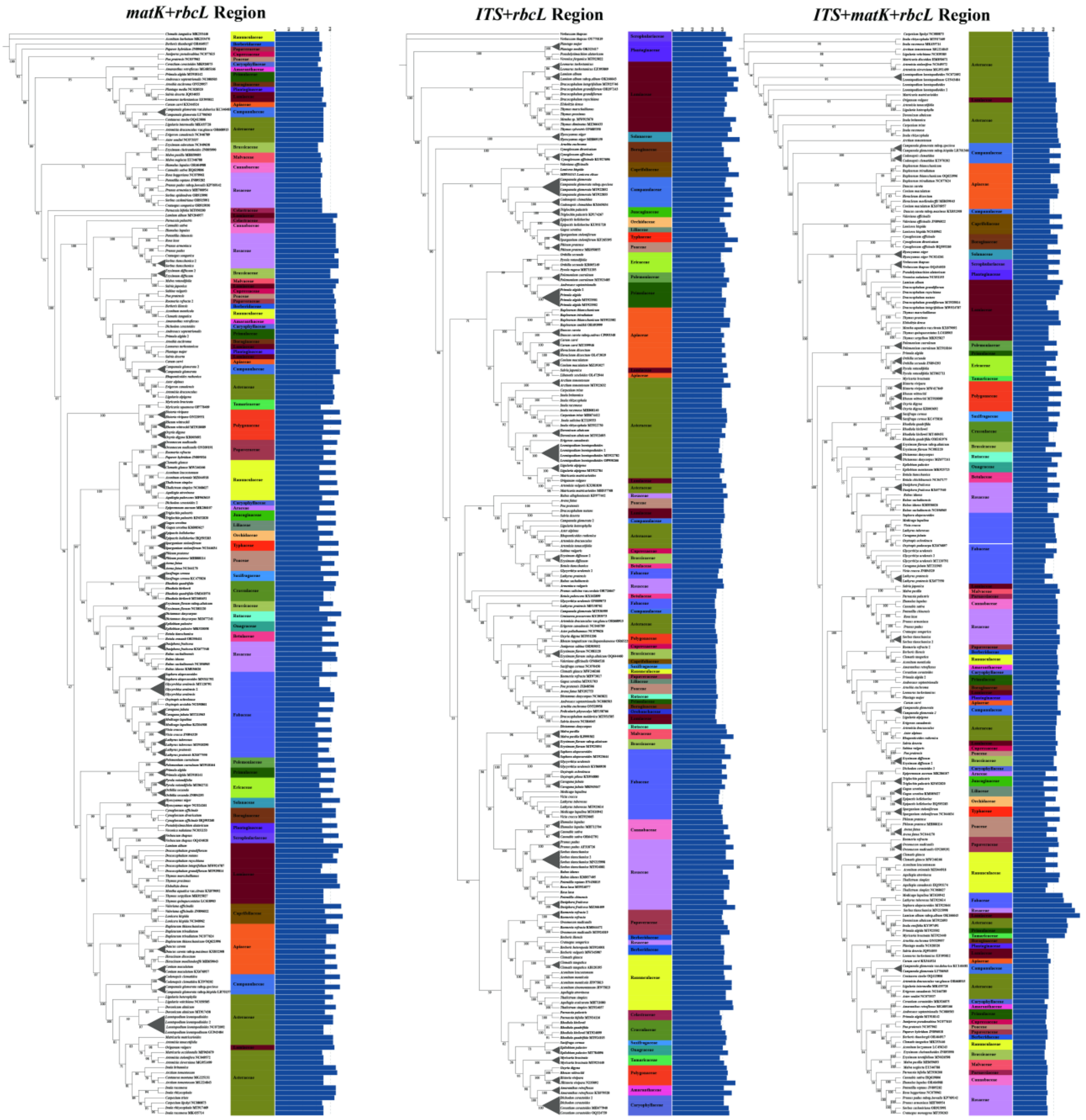

Figure S1. The Maximum likelihood (ML) trees of the 109 samples of medicinal plants(101 species). Tree were constructed based on the ITS, *rbcL*, *matK* regions, as well as the following combinations: ITS+*matK*, ITS+*rbcL*, *matK*+*rbcL and* ITS+*matK*+*rbcL*. Bootstrap values are shown on the branches, ranging from 50% to 100%. Species names without NCBI number represent sequences generated in this study, while the other species sequences with NCBI number were downloaded via BLASTn from NCBI. The blue rectangles represent the GC content for each sequence.
